# Altered sphingolipid pathway in SARS-CoV-2 infected human lung tissue

**DOI:** 10.3389/fimmu.2023.1216278

**Published:** 2023-10-04

**Authors:** Rabisa J. Khan, Sierra L. Single, Christopher S. Simmons, Mohammad Athar, Yuelong Liu, Sandeep Bodduluri, Paul V. Benson, Kayla F. Goliwas, Jessy S. Deshane

**Affiliations:** ^1^ Department of Medicine, Division of Pulmonary, Allergy, and Critical Care Medicine, University of Alabama at Birmingham, Birmingham, AL, United States; ^2^ University of Alabama at Birmingham Heersink School of Medicine, Birmingham, AL, United States; ^3^ Department of Dermatology, University of Alabama at Birmingham, Birmingham, AL, United States; ^4^ Department of Pathology, University of Alabama at Birmingham, Birmingham, AL, United States

**Keywords:** sphingolipid signaling, SARS-CoV-2, COVID-19, COVID-19 convalescence, lung structural remodeling

## Abstract

**Introduction:**

The SARS-CoV-2 mediated COVID-19 pandemic has impacted millions worldwide. Hyper-inflammatory processes, including cytokine storm, contribute to long-standing tissue injury and damage in COVID-19. The metabolism of sphingolipids as regulators of cell survival, differentiation, and proliferation has been implicated in inflammatory signaling and cytokine responses. Sphingosine-kinase-1 (SK1) and ceramide-synthase-2 (CERS2) generate metabolites that regulate the anti- and pro-apoptotic processes, respectively. Alterations in SK1 and CERS2 expression may contribute to the inflammation and tissue damage during COVID-19. The central objective of this study is to evaluate structural changes in the lung post-SARS-CoV-2 infection and to investigate whether the sphingolipid rheostat is altered in response to SARS-CoV-2 infection.

**Methods:**

Central and peripheral lung tissues from COVID-19+ or control autopsies and resected lung tissue from COVID-19 convalescents were subjected to histologic evaluation of airspace and collagen deposisiton, and immunohistochemical evaluation of SK1 and CERS2.

**Results:**

Here, we report significant reduction in air space and increase in collagen deposition in lung autopsy tissues from patients who died from COVID-19 (COVID-19^+^) and COVID-19 convalescent individuals. SK1 expression increased in the lungs of COVID-19^+^ autopsies and COVID-19 convalescent lung tissue compared to controls and was mostly associated with Type II pneumocytes and alveolar macrophages. No significant difference in CERS2 expression was noted. SARS-CoV-2 infection upregulates SK1 and increases the ratio of SK1 to CERS2 expression in lung tissues of COVID-19 autopsies and COVID-19 convalescents.

**Discussion:**

These data suggest an alteration in the sphingolipid rheostat in lung tissue during COVID-19, suggesting a potential contribution to the inflammation and tissue damage associated with viral infection.

## Introduction

Severe acute respiratory syndrome COVID-19, caused by the novel coronavirus (SARS-CoV-2), continues to serve as a global health emergency, estimating over 761 million cases and 6.8 million deaths as of March 2023 (1). Acute and severe lung injury and respiratory failure have significantly contributed to mortality of many immunocompromised individuals infected with COVID-19 (2, 3). Viral infection-induced lung tissue damage and hyper-inflammatory and immune-mediated responses contribute to the pathogenesis of long-standing lung tissue injury (2, 4, 5). Disease severity is heightened in the elderly and immunocompromised individuals, where persistent inflammation leads to severe tissue damage (3). Some COVID-19 convalescent individuals continue to experience the long-term effects from viral infection including multisystem injury referred to as long COVID (5, 6).

Evidence suggests that hyper-activation of the immune system and an uncontrolled release of cytokines, known as cytokine release syndrome or “cytokine storm,” underlies COVID-19 related respiratory failure and death (3, 7, 8). In SARS-CoV-2 infection, the virus infects the respiratory epithelial tissue and activates local innate immune cells to release proinflammatory cytokines and other chemokines (3, 9). These proinflammatory cytokines and chemokines induce aberrant inflammatory signaling pathways via receptors on immune cells and tissue structural cells. This then leads to the recruitment of additional innate immune cells and activation of adaptive immune cells from peripheral tissues to produce sustained inflammatory cytokine release (3, 7, 8). This further aggravates lung and epithelial damage and contributes to acute respiratory distress syndrome. Intercellular presence of the virus has also been shown to disrupt the endothelial membranes and lead to complications such as pulmonary edema, intravascular coagulation and multiorgan failure (10). This disruption in the intercellular junctions allows an influx of circulating lymphocytes, perivascular inflammation, and overall severe lung damage (10). Inflammation induced dysregulation of alveolar epithelial regeneration promotes the hyperproliferation and fibrosis seen with this virus (11, 12). Type II pneumocytes proliferate to initiate the reparative response post SARS-CoV-2 infection and the defective alveolar type II pneumocyte differentiate into alveolar type I pneumocytes resulting in impaired lung regeneration (11–13). Several studies have linked a destructive hyperinflammatory response to COVID- pneumonia and the high mortality associated with SARS-CoV-2 infection (8, 9, 13).

Sphingolipids are a major class of complex eukaryotic phospholipids that are key structural components of cell membranes involved in the regulation of cell survival, proliferation, and the inflammatory response (14). This family of lipids includes sphingosine, ceramide, sphingosine-1-phosphate (S1P) and ceramide-1-phosphate, as well as many others (15). Ceramide, the central pro-apoptotic molecule produced by various ceramide synthase enzymes (CERS 1-6), regulates epithelial and endothelial apoptosis in lung tissue (16). Ceramide is then converted to sphingosine, a precursor to sphingosine-1 phosphate (S1P). Sphingosine is converted to S1P by sphingosine kinases 1 or 2 (SK1 or SK2) (16). S1P acts on S1P receptors to induce inflammation and promote pro-survival processes (14).

Sphingolipid metabolism has been implicated in inflammatory signaling and cytokine responses (14). The sphingolipid rheostat also regulates cell survival, viral replication, and maintenance of endothelial integrity (10). Dysregulation of the sphingolipid rheostat, particularly the S1P pathway, is involved in the cytokine storm and hyperinflammatory response seen in a plethora of lung pathologies, such as pulmonary fibrosis, pulmonary arterial hypertension, lung cancer, and viral infection of the lung (14). This dysregulation of the immune system along with damage to membrane barriers and surrounding tissues is what causes the critical symptoms of this disease, leading to increased mortality in the elderly and immunocompromised (10).

Sphingosine-kinase-1 (SK1) and ceramide-synthase-2 (CERS2) generate metabolites that regulate these anti- and pro-apoptotic processes, respectively (10). Increased expression of SK1 mediated synthesis of S1P was seen in influenza and respiratory syncytial virus (RSV), suggesting promotion of viral entry, replication, and production of viral proteins (17, 18). Additionally, an overexpression of SK1 in these disease states demonstrated a decrease in ceramide content, demonstrating their antagonistic effects (19). Alterations in SK1 and CERS2 expression may correlate with the pathogenesis of lung injury in COVID-19 affected individuals. The death/survival balance (ceramide/S1P) balance between these two antagonistic signaling pathways is of great interest in SARS-CoV-2 infection, as SK1 driven production of S1P may be contribute to the hyper-inflammatory, cytokine storm that leads to ARDS (19). Thus, modulation of this pathway may lead to therapeutic effects in the future.

The objectives of this study are to examine alterations to the sphingolipid rheostat; specifically SK1 and CERS2 expression, and how that may contribute to structural lung changes, and examine its’ overall impact on lung tissue health in response to the SARS-CoV-2 infection.

## Methods

### Sample acquisition

Formalin-fixed paraffin-embedded lung tissue sections were obtained from SARS-CoV-2 infected (died from COVID-19) and uninfected (control) autopsy samples with the help of the University of Alabama at Birmingham Tissue Biorepository facility. De-identified, remnant surgical specimen were obtained from lobectomy and wedge resection surgeries performed at the University of Alabama at Birmingham. 5 tissue specimen were obtained from patients with no history of SARS-CoV-2 infection and 8 tissue specimen were obtained from patients who had previously tested positive for SARS-CoV-2 and cleared the infection (convalescence period detailed in **Supplemental Table 1**). Tissue microarrays using 2 mm tissue cores (2 cores per sample) were generated from these samples. This study was approved by the University of Alabama at Birmingham Institutional Review Board (IRB-#300006867 and IRB-#300003092) and conducted following approved guidelines and regulations.

### Immunohistochemistry and histological staining

5 micron sections were stained with hematoxylin and eosin (H&E) to evaluate tissue morphology and air space and Masson’s trichrome stain to evaluate degree of collagen deposition. Immunohistochemistry was performed to detect SK1 (1:200, LS Bio, Seattle, WA, USA), CERS2 (1:1200, OTI3D9, OriGene, Rockville, MD, USA), S1PR1 (1:250, 8B7.1, MilliporeSigma, Burlington, MA, USA), or S1PR2 (1:200, Proteintech, Rosemont, IL, USA) using the Dako Envision + Dual Link secondary detection kit (Agilent Technologies, Santa Clara, CA) and chromogens 3,3’-Diaminobenzidine (DAB; brown; SK1, CERS2, and S1PR1 staining) or AEC (3-amino-9-ethylcarbazole) HRP Substrate (red; S1PR2 staining) following antigen retrieval (SK1, S1PR1 & S1PR2:10 mM citrate buffer (pH 6, Biogenex, San Ramon, CA, USA), and CERS2: Tris/EDTA buffer (pH9, Agilent, Santa Clara, CA, USA)). Representative no antibody staining controls shown in **Supplemental Figure 2**. Additionally, SK1 (secondary: anti-rabbit AlexaFluor594, Invitrogen,Waltham, MA, USA) and SARS-CoV-2 Nucleocapsid protein (1:1000, clone 05, Sino Biological, Wayne, PA, USA; secondary: anti-mouse AlexaFluor647, Invitrogen) were evaluated via immunofluoresent staining following citrate buffer antigen retrieval.

### Image analysis

An in-house image processing pipeline was developed to compute % air space and % collagen from photomicrographs. The pipeline includes a semi-automated thresholding operation that initially provided color-based segmentation maps by an optimal separation of red, blue, and green channels for airspace computation and hue, saturation, and value for collagen estimation. These identified thresholds were validated in a subset of images by comparing values with manual estimations and were further adjusted to minimize error. The chosen thresholds were then applied to all images to compute % air space and % collagen. The image processing pipeline was developed in MATLAB R2021a (Mathworks, Natick, MA).

Air space, defined as average % air space per tissue, was determined from photomicrographs (200x) of H&E-stained histologic sections of lung samples from autopsies (minimum of 20 randomly selected regions were photomicrographed per tissue, minimum of 7 tissues per group). Photomicrographs were averaged to compute average air space per tissue. Collagen, defined as average % collagen per tissue, was determined from photomicrographs (200x) of Masson’s trichrome-stained histologic sections of lung samples from autopsies (minimum of 20 randomly selected regions were photomicrographed per tissue, minimum of 5 tissues per group). Photomicrographs were averaged to compute average collagen deposition per tissue.

SK1^+^ and CERS2^+^ cells were quantitated in a minimum of 20 photomicrographs (400x, randomly selected regions) per tissue using QuPath automated cell detection. The average % positive per tissue was determined. All sections were imaged with an EVOS cell imaging microscope and photomicrographs were saved as .tiff images.

### Statistical analysis

Statistical analysis was performed using GraphPad Prism software (La Jolla, CA, USA). Data are presented as mean ± standard error of the mean unless otherwise indicated. A *p-*value less than 0.05 was considered to be statistically significant. A two-tailed unpaired Student’s *t*-test was used to evaluate statistical difference between two groups. One way ANOVA with Sidak’s multiple comparison testing was utilized to evaluate statistical difference for data with more than two groups.

## Results

**Figure 1:**
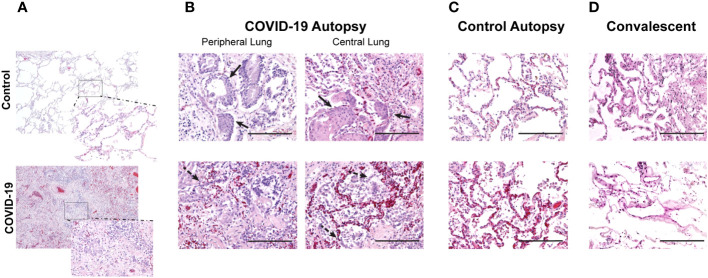
Changes in air space in central and peripheral lung samples from COVID-19 and control autopsies, and convalescent lung samples. **(A)** Comparison of lung histologic cross-sections from control and COVID-19 autopsy; 40x magnification with 200x magnification inset. **(B–D)** Representative photomicrographs of hematoxylin & eosin-stained lung tissue from COVID-19 autopsy **(B)**, control autopsy **(C)**, and convalescent lung tissue **(D)**. 200x magnification (scale bar: 200 microns). Filled arrow: metaplasia of type II pneumocytes; dashed arrow: hyperplasia of type II pneumocytes.

As SARS-CoV-2 infections are known to cause extensive lung tissue damage, we first evaluated overall lung architectural changes in hematoxylin & eosin (H&E) stained lung cross-sections obtained from autopsies. Samples were obtained from autopsies performed on patients who died with COVID-19 (COVID-19^+^) and those who had not been infected with COVID-19 (control). When comparing these study groups, we first noted loss of air space in COVID-19^+^ lung specimen (**Figures 1A, B**). Pathologic changes in COVID-19 lungs showed alveolar fibroblast proliferation, type II pneumocyte hyperplasia and metaplasia, and mild to moderate interstitial lymphocytic infiltrate, as seen in **Figure 1A** (COVID-19 autopsy lung). Peripheral and central lung sections from COVID-19 autopsies appeared histologically similar to each other with alveolar fibroblast proliferation with some areas of collagen deposition, organizing hyaline membranes, abundant type II pneumocyte hyperplasia with squamous metaplasia, and occasional bronchiolar metaplasia with moderate to focally increased lymphocytic infiltrates (**Figure 1B**). These changes are all consistent with organizing diffuse alveolar damage.

**Figure 2:**
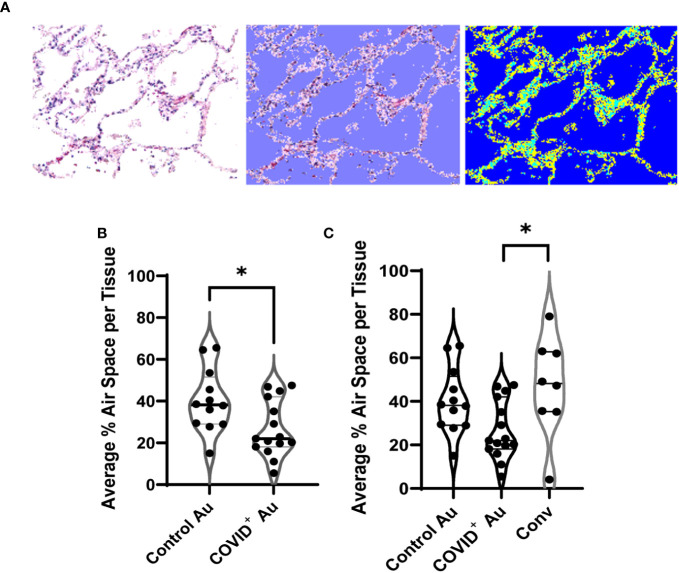
Quantification of air space in histologic cross sections. **(A)** Example of the custom MATLAB script used to quantify airspace, with the blue (right) indicating the air space measured by the script. **(B, C)** Comparison of air space between control and COVID^+^ autopsies **(B)**, and convalescent lung samples **(C)**. n=8-15. *p<0.05.

As long-term effects due to COVID-19 are often seen in COVID-19 convalescents, we performed detailed evaluation of air space in both peripheral and central lung samples from COVID-19 (**Figure 1B**) and control autopsies (**Figure 1C**) as well as remnant surgical lung specimens from COVID-19 convalescents (**Figure 1D**). Using photomicrographs of H&E stained lung histologic cross-sections and tissue microarrays, both generated from FFPE blocks of these samples, we quantitated the air space using an in house developed MATLAB script, as shown in **Figure 2A**. For this analysis, 20 photomicrographs of each histologic cross-section were analyzed and the average air space per tissue was determined. The average air space per tissue was significantly reduced in the COVID-19 autopsy group compared to the control autopsy group (**Figure 2B**). Further, the average air space in COVID-19 autopsy lung specimen was significantly lower than the COVID-19 convalescent specimen (*p<0.05); notably average air space in convalescents was not significantly different than the controls (**Figure 2C**).

**Figure 3:**
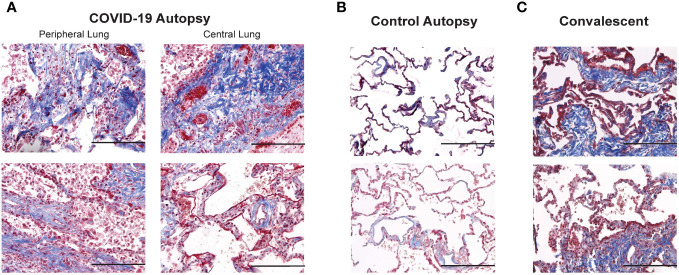
Comparison of collagen deposition in COVID-19 and control autopsies, and convalescent lung samples **(A–C)** Photomicrographs of Masson's trichrome stained peripheral and central lung samples from COVID-19 autopsies **(A)**, control autopsies **(B)** and convalescent lung samples **(C)**; 200 x magnification (scale bar: 200 microns).

**Figure 4:**
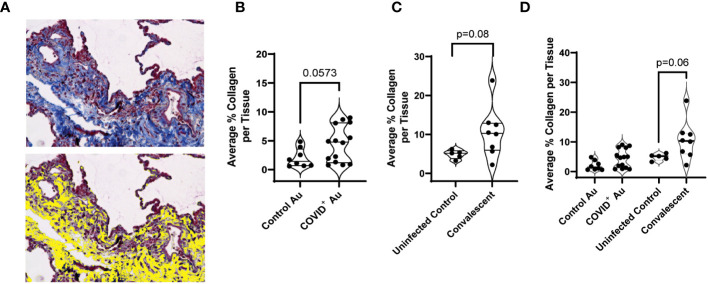
Quantification of collagen deposition. **(A)** Photomicrographs were analyzed using a custom MATLAB script to quantify collagen (blue) staining, with yellow indicating collagen-rich areas (bottom) **(B–D)** Comparison of collagen deposition between control and COVID^+^ autopsy samples **(B)**, uninfected control and convalescent lung samples **(C)**, and control, convalescent, and COVID^+^ autopsies **(D)**. n=5-14.

As changes in lung stiffness and fibrotic scars are often reported in lung injury associated with COVID-19, we assessed changes in collagen deposition in lung histologic sections from COVID-19^+^ and uninfected control autopsies, as well as remnant surgical specimen from COVID-19 convalescents. Lung tissue cross sections were stained with Masson’s trichrome to assess changes in collagen structure, specifically evaluating differences in collagen deposition between the study groups. Photomicrographs of histologic cross-sections stained with Masson’s trichrome were acquired to compare COVID-19 autopsies (**Figure 3A**), control autopsies (**Figure 3B**) and COVID-19 convalescent specimen (**Figure 3C**). 20 photomicrographs per sample were acquired and a MATLAB script was used to quantify the average percentage of collagen (blue) present per tissue, as shown in **Figure 4A** where the blue collagen in the top image is recognized by the custom MATLAB script and shown by the yellow signal in the bottom image. An increase in collagen deposition (p=0.0573) was found in the COVID-19^+^ autopsy lung specimen (**Figure 4B**) compared to the control autopsy. Average % collagen deposition per tissue in remnant lung tissue from COVID-19 convalescents was increased but not significantly different from uninfected remnant lung tissue controls (**Figure 4C**). Comparison of collagen deposition in all groups showed that COVID-19 convalescents had the highest collagen deposition compared with diseased and control autopsies as well as the uninfected tissue controls (**Figure 4D**). The COVID-19 convalescent tissue with highest collagen deposition also had the lowest average airspace.

**Figure 5:**
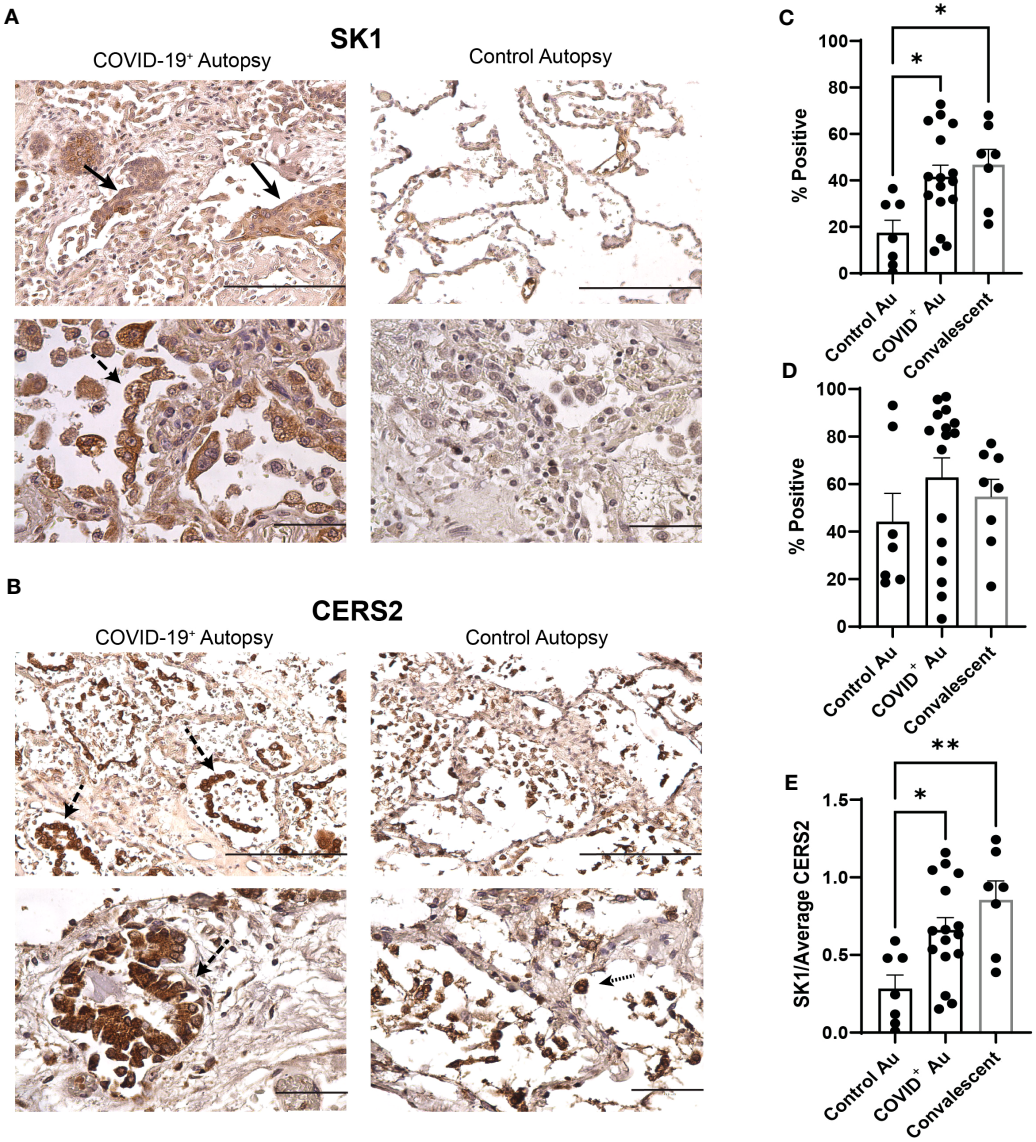
Alterations in sphingolipid metabolism within COVID-19 lungs. **(A, B)** Photomicrographs of COVID-19^+^ and control lung autopsy samples stained for SK1 **(A)** & CERS2 **(B)**. **(C)** Comparison of SK1 expression between control, COVID^+^, & convalescent samples (n=7-16). **(D)** Comparison of CERS expression between control, COVID^+^, & convalescent samples (n=7-16). **(E)** Ratio of SK1:CERS expression in control, COVID^+^, & convalescent samples (n=7-16). Filled arrows denote squamous metaplasia; dashed arrows denote type II pneumocyte hyperplasia; dotted arrows denote alveolar macrophages (scale bar: 200 microns (top images in each panel) or 100 microns (bottom images in each panel). *p≤0.05 **p<0.01.

We next assessed if alterations in the sphingolipid rheostat may contribute to hyperproliferative responses and reduced airspace/structural changes noted in the COVID-19 autopsies. The expression of SK1 and CERS2 was assessed in control, COVID-19^+^, and convalescent samples using immunohistochemistry. Representative photomicrographs of SK1 and CERS2 staining in lung tissues from both COVID-19^+^ and control autopsies are shown in **Figure 5A**. Compared to control autopsies, SK1 expression in the COVID-19^+^ autopsies was increased in alveolar type II pneumocytes, many of which showed squamous metaplasia (**Figure 5A**). Scattered single alveolar cells (possibly identified as alveolar macrophages) stained positive for SK1. CERS2 staining was prominent in activated type II pneumocytes in COVID-19^+^ autopsies (**Figure 5B**). Control autopsy tissues showed staining in groups of single cells in the alveolar spaces, likely representing alveolar macrophages. Quantitation of expression of SK1 and CERS2 by QuPath analyses showed significant increase in % positive cells expressing SK1 in COVID-19^+^ autopsies and convalescent samples compared to control autopsies (**Figure 5C**). Additionally, we evaluated co-expression of SK1 and nucleocapsid protein of SARS-CoV-2 in these lung sections. As shown in **Supplemental Figure 1**, immunofluorescence analyses showed SK-1^+^ nucleocapsid lung cells in COVID-19^+^; not all SK-1^+^ cells were expressing the viral protein. When SK1 expression was evaluated in central and peripheral lung sections of COVID-19^+^ autopsies, % SK1^+^ cells were significantly higher in central lung control autopsies (**Supplemental Figure 3A**). Despite histological evaluation showing CERS2 expression in different cell populations (active type II pneumocytes vs alveolar macrophages), the percentage of CERS2 positive cells was not different between the study groups (**Figures 5B, D**; **Supplemental Figure 3B**). The ratio of SK1 to CERS2 expression was increased in COVID-19^+^ and COVID-convalescent samples compared to control autopsies (**Figure 5E**), with a significant increase in central lung sections of the COVID-19^+^ autopsy (**Supplemental Figure 3C**). Additionally, we included evaluations of expression of SK1, CERS2 and SK1/CERS2 ratio in additional lung sections from control autopsies that exhibited non-COVID-19 pneumonia and diffused alveolar damage from patients with non-COVID-19 related ARDS to determine if the alteration of the sphingolipid rheostat was specific to COVID-19. As shown in **Supplemental Figures 3D–F**, expression of SK1 was altered specifically in COVID-19^+^ autopsies compared to these controls.

As SK1 expression and SK1/CERS2 ratios were increased in COVID-19^+^ autopsies, we then evaluated if lipid signaling through the sphingolipid receptors would be enhanced in these autopsies. As the anti-apoptotic lipid S1P signals through S1PR1 and S1PR2, we qualitatively evaluated their expression in these autopsies. As shown in **Supplemental Figure 4A**, S1PR1 expression was localized to submucosal gland like structures in both control and COVID-19^+^ autopsies with no notable difference in expression. Expression of S1PR2 was more robust in the squamous metaplasia in the COVID-19^+^ autopsies (**Supplemental Figure 4B**). Interestingly, in COVID-19 convalescents, macrophage like cells in the alveolar lining were S1PR2^+^. Together, these data suggest that sphingolipid signaling may be altered due to SARS-CoV-2 infection.

## Discussion

One of the hallmark features of the COVID-19 is the hyperactivation of the immune system and subsequent damage to lung tissue, ultimately leading to a reduction in air exchange in the lungs (2, 5, 9). While novel mechanisms of infection and signaling pathways induced by the novel SARS-CoV-2 continue to be investigated, it has been well established that the hyper-inflammatory response to this virus leads to lung tissue injury (3, 4, 8). Accumulation of fluid and cellular debris within air spaces, as well as damage to the cells lining the airways and alveoli, are thought to contribute to the loss of elasticity and increased resistance to air flow associated with COVID-19^+^ individuals (3, 4, 8). This aberrant activation of the immune system may contribute to the reduction of air space and increase in collagen deposition seen in the lung tissues of those infected with this virus. Emerging evidence also suggests that COVID-19 may alter sphingolipid metabolism, a complex process that involves the synthesis, breakdown, and interconversion of sphingolipids and their metabolites in various cell types (14, 20). Bioactive sphingolipids ceramide and S1P of the sphingolipid pathway serve as signaling counterparts, maintaining a homeostatic balance within the body to regulate cell survival (19, 21). Alterations in this sphingolipid signaling observed in our study suggest a potential contribution to inflammation and lung tissue damage often seen with COVID-19. Our observed changes in the enzymes involved in sphingolipid regulation complements recently published studies that have shown altered lipid metabolism in serum sphingolipids in patients infected with SARS-CoV-2 and murine infection models (22, 23). Future studies delineating COVID-19 associated alterations in sphingolipid metabolism are important for developing new therapies targeting the pro-survival sphingolipid enzymatic pathway in the context of viral infections.

One of the ways in which COVID-19 can impact the respiratory system is by reducing the amount of air space in the lungs. Several factors including inflammation and damage to airway and alveoli lining, as well as accumulation of fluid and cellular debris within the air spaces, contribute to air space reduction (24). Previous studies have demonstrated loss of alveolar basement membrane integrity with a decrease in the lacunar space of SARS-CoV-2 infected lungs compared to healthy lung tissue (9, 24). In our study, we compared remnant lung tissue samples from uninfected individuals (control) and COVID-19 convalescents, COVID-19 infected autopsies (COVID-19^+^) and control autopsies for changes in overall lung architecture. The significant loss of air space in COVID-19^+^ lung specimens compared to controls highlights the damage to cells within the lung. The pathologic proliferation of fibroblasts, type II pneumocyte hyperplasia and metaplasia along with lymphocytic infiltration in the COVID-19 autopsy lung may have contributed to the diffused alveolar damage and loss of air space. This loss in air space can negatively impact gas exchange and ultimately lead to respiratory distress. Interestingly, when comparing central and peripheral lung tissue samples from COVID-19^+^ autopsies to remnant lung specimens from COVID-19 convalescents, a significant decrease in average air space was observed within COVID-19^+^ yet individuals who recovered from COVID-19 did not show this tissue response. While no significant changes were observed in air spaces between central and peripheral COVID-19^+^ lung tissues, the reduction in lacunar spaces within the COVID-19^+^ lung tissue when compared to the control lung tissue highlights the alveolar damage present with COVID-19 infection. Cell damage and loss of lacunar space for gas exchange is further complicated when interstitial edema accumulates, impairing the ability of the remaining alveoli and contributing to respiratory distress, like the process of alveolar flooding associated with acute respiratory distress syndrome (2, 25).

Additionally, damage to alveolar architecture and reduction in air space in the lung often leads to dysregulated tissue repair and fibrosis (24). In our studies, an increase in collagen deposition was found in COVID-19^+^ lung tissue when compared to control lung samples; this is consistent with dysregulated tissue repair that results from SARS-CoV-2 infection. A moderate increase in collagen deposition was noted within lung tissue samples from COVID-19 convalescents when compared to both COVID-19^+^ and control lung tissue. This increase in collagen deposition in convalescent lung samples may reflect the advanced consequent tissue fibrosis often seen in COVID-19 convalescents. Increased fibrosis in COVID-convalescent patients could explain the long-standing effects that “long- COVID” has on many patients.

Our investigations of sphingolipid signaling associated with COVID-19 is important as sphingolipids and their metabolites play critical roles in various cellular processes, including the regulation of the immune system, inflammation, and cell death (14, 26). Sphingolipids are also involved in maintaining the integrity of the cellular membrane and are crucial for the normal functioning of various organs, including the lungs, liver, and brain (14, 15). Emerging evidence suggests that severity of COVID-19 disease may be associated with altered S1P levels (27), impairing endothelial barrier function, and leading to lung injury and respiratory distress (10). In our study, the expression of SK1, an enzyme that generates anti-apoptotic S1P, and CERS2, regulator of pro-apoptotic processes, was analyzed in both diseased, convalescent and control lung tissues. The increase in SK1 expression in type II pneumocytes in COVID-19^+^ are consistent with the hyper-proliferative reparative response in the epithelium post-injury reported with SARS-CoV-2 infection and often associated with COVID-pneumonia (13, 24, 28). Alveolar type II pneumocytes as defenders of the alveolus are critical to repairing COVID-19 lung injury. The differential CERS2 expression, with an enhanced signal in type II pneumocytes in COVID-19 autopsy, is potentially due to apoptotic process initiated by viral entry and infection through ACE2 or other viral receptors in the epithelium. In contrast, CERS2 expression in alveolar macrophages in control autopsies may reflect the apoptotic cell death commonly associated with death of the tissue; increase in expression of both these enzymes are consistent with activation of sphingolipid metabolism with SARS-CoV-2 infection. The observed increase in ratio of SK1 to CERS2 expression in COVID-19 and convalescent samples compared to the control suggests alterations in the sphingolipid rheostat with COVID-19 infection and a potential role for this pathway in survival and turnover of both structural and immune cells in the lungs post-infection. While SK1 expression is largely associated with non-lymphocytes, the levels of anti-apoptotic lipid S1P are known to regulate lymphocyte trafficking (29, 30). Additionally, S1P levels can influence fibroblast activation and proliferation (16, 31). Our studies have limitations, as the sphingolipids and their metabolites were not quantitated in these patient samples due to COVID related restrictions for mass spec analyses. Our immunohistochemical investigations have also limited us from assessing S1P levels. Our evaluations of S1PR1 and S1PR2 suggest that the SK1 induction and downstream signaling may have involved S1PR2 in COVID-19^+^ autopsies and not S1PR1. It is possible that SK1-induced S1P signaling may have influenced alveolar fibroblast proliferation and lymphocyte infiltration that we observed in COVID-19^+^ lungs. Observed increases in pro-survival SK1 relative to pro-apoptotic CERS2 may provide insights into the persistent inflammation and fibrosis and unravel mechanisms underlying the COVID-19 disease progression. Future studies are warranted to assess changes in sphingolipid metabolism to provide more insights on the role of the sphingolipid pathway in COVID-19. Understanding specific alterations in the sphingolipid rheostat could lead to the identification of new therapeutic targets (32), help monitor disease progression and severity, and improve our understanding of the disease pathogenesis.

## Conclusions

In conclusion, SARS-CoV-2 infection negatively affects lung architecture and the sphingolipid signaling pathway. Average loss of air space and increase in collagen deposition was identified in COVID-19 autopsies and convalescent lung samples, respectively, when compared to control uninfected lung tissues. Additionally, we identified that both SK1 and the ratio between SK1 and CERS2 (pro-apoptotic) enzyme expression in lung tissue is increased after SARS-CoV-2 infection. Alteration in the sphingolipid rheostat in which SK1 (pro-survival) is upregulated, compared to its counterpart ceramide (pro-apoptotic), in lung tissue during COVID-19 may be contributing to the overall inflammation and tissue damage seen with the SARS-CoV-2 infection. In the future, understanding sphingolipid metabolism alterations in COVID-19 disease may help in the development of new therapeutic strategies in the future to mitigate its adverse effects.

## Data availability statement

Upon request the raw data supporting the conclusions of this article will be made available by the authors, without undue reservation.

## Ethics statement

The studies involving humans were approved by University of Alabama at Birmingham Institutional Review Board. The studies were conducted in accordance with the local legislation and institutional requirements. The participants provided their written informed consent to participate in this study.

## Author contributions

RK and SS contributed equally to this study and are listed as co-first authors. Both were involved in execution of experiments, data collection, data analysis, and manuscript and figure preparation. CS and YL were involved in data collection and data analysis. MA was involved in manuscript editing and gave critical insights. SB generated MATLAB scripts and was involved in customizing the scripts for data analyses. SB was involved in writing methods section of the manuscript. PB was involved in all aspects related to pathological and histological evaluations of COVID-19^+^ and control autopsies and contributed to manuscript preparation. KG and JD were involved in experimental design and oversight, data interpretation, and manuscript and figure preparation. All authors contributed to the article and approved the submitted version.
