# Supplementary material for: Altered sphingolipid pathway in SARS-CoV-2 infected human lung tissue
Source: Front Immunol. 2023 Oct 4;14:1216278. doi: 10.3389/fimmu.2023.1216278 (PMC10585362; doi:10.3389/fimmu.2023.1216278)
Supplement: Supplementary file 1 [file DataSheet_1.zip › Supplementary Material/Supplementary Figure 1.pdf]

Supplemental Figure 1

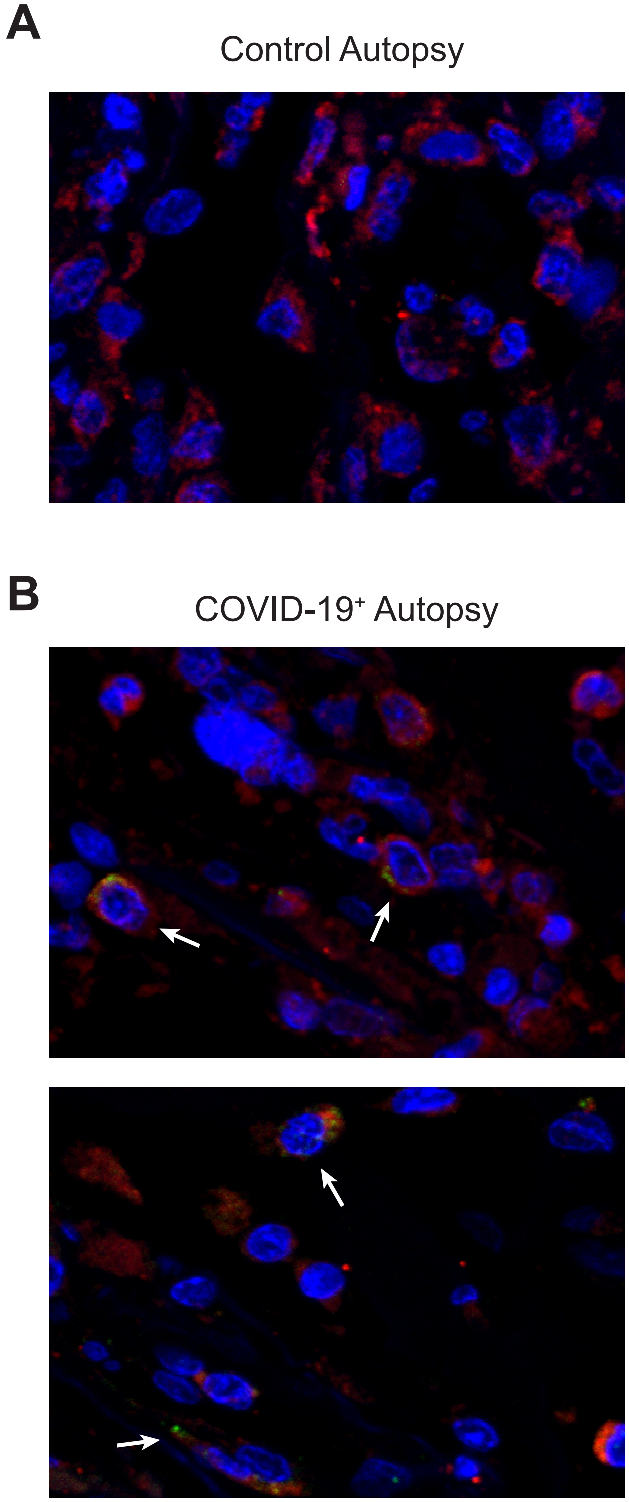

Supplemental Figure 1. SK1 expression and COVID-19 Nucleocapsid Protein. SK1 expression (red) is observed in both control and COVID-19 autopsy lung specimen, with expression overlapping with COVID-19 nucleocapsid protein (green) in COVID-19 autopsy lung specimen.
