# Supplementary material for: Altered sphingolipid pathway in SARS-CoV-2 infected human lung tissue
Source: Front Immunol. 2023 Oct 4;14:1216278. doi: 10.3389/fimmu.2023.1216278 (PMC10585362; doi:10.3389/fimmu.2023.1216278)
Supplement: Supplementary file 1 [file DataSheet_1.zip › Supplementary Material/Supplementary Figure 2.pdf]

Supplemental Figure 2

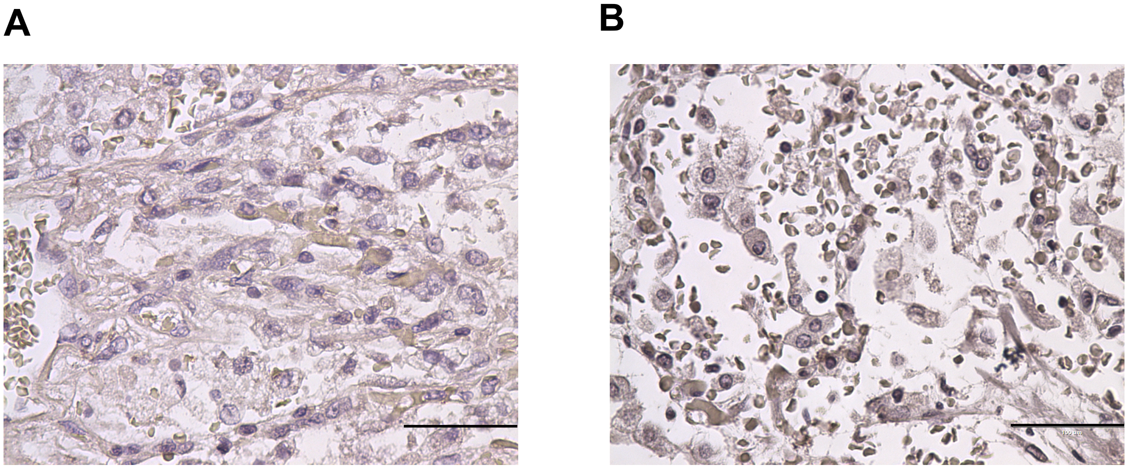

Supplemental Figure 2. Immunohistochemistry staining controls. **A.** No antibody control following pH6 antigen retrieval. **B.** No antibodycontrol following pH10 antigen retrieval. (Scale bar: 100 microns).
