# Supplementary material for: Altered sphingolipid pathway in SARS-CoV-2 infected human lung tissue
Source: Front Immunol. 2023 Oct 4;14:1216278. doi: 10.3389/fimmu.2023.1216278 (PMC10585362; doi:10.3389/fimmu.2023.1216278)
Supplement: Supplementary file 1 [file DataSheet_1.zip › Supplementary Material/Supplementary Figure 3.pdf]

Supplemental Figure 3

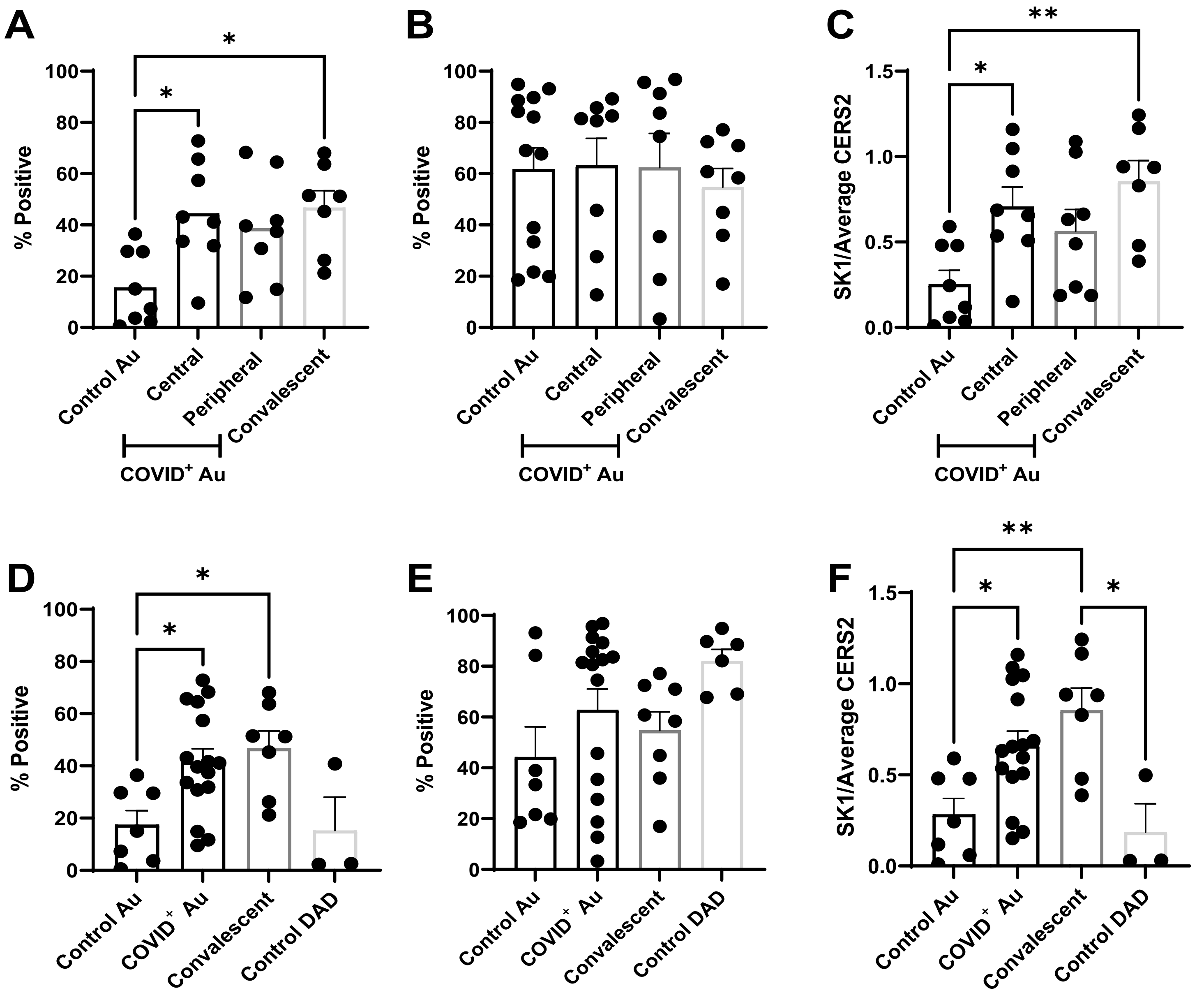

Supplemental Figure 3. Sphingolipid Mediators in Central & Peripheral COVID-19 Autopys and Non-COVID-19 associated Diffuse Alveolar Damage. **A-C**. SK1 (**A**), and CERS2 (**B**) expression and the ratio of SK1/CERS2 (**C**) when COVID-19 autopsy data is subgrouped by lung site. **D-F**. SK1 (**D**), and CERS2 (**E**) expression and the ratio of SK1/CERS2 (**F**) in control autopsy, COVID-19 autopsy, and COVID-19 convalescent lung samples compared to non-COVID-19 associated diffuse alveolar damage (with ARDS diagnosis). n-3-16. * p≤0.05 **p≤0.01
