# Supplementary material for: Altered sphingolipid pathway in SARS-CoV-2 infected human lung tissue
Source: Front Immunol. 2023 Oct 4;14:1216278. doi: 10.3389/fimmu.2023.1216278 (PMC10585362; doi:10.3389/fimmu.2023.1216278)
Supplement: Supplementary file 1 [file DataSheet_1.zip › Supplementary Material/Supplementary Table 1.PDF]

Supplemental Table 1: Convalescent Lung Samples

Convalesent Sample	Convalescence Period (Prior to Resection; Days)	Sex	Age
1	28	Female	56
2	77	Male	68
3	104	Female	73
4	75	Male	46
5	450	Female	67
6	450	Male	59
7	443	Female	75
8	148	Female	40
9	201	Male	62
